# Palliative care for children with cancer in Cuba: a narrative review and adaptation of the World Health Organization integration model

**DOI:** 10.3389/fped.2026.1908007

**Published:** 2026-08-18

**Authors:** Mariuska Forteza Sáez, Maria del Carmen Llantá Abreu, Yolainy Romero Rodríguez, Dayne Clarivel Quintero Vázquez

**Affiliations:** 1 Pediatric Oncology Service, Institute of Oncology and Radiobiology (INOR), Havana, Cuba; 2 Psycho-Oncology Service, Institute of Oncology and Radiobiology (INOR), Havana, Cuba

**Keywords:** childhood cancer, Cuba, palliative care integration, pediatric palliative care, quality of life, WHO model

## Abstract

**Background:**

Palliative care for children with cancer represents an urgent health priority in Cuba. The country has a universal and free health system with national coverage, but the systematic provision of specialized pediatric palliative care (PPC) in oncology remains heterogeneous, with significant gaps across the eight pediatric oncology reference centers.

**Objective:**

To describe the state of PPC development for children with cancer in Cuba, analyze it through the lens of the simultaneous integration model proposed by the World Health Organization (WHO), and identify gaps and opportunities for strengthening. This review is intentionally limited to pediatric oncology; PPC needs of children with non-oncological life-limiting conditions fall outside its scope.

**Methods:**

Narrative review of scientific literature published between 2000 and 2024, normative documents of the Cuban Ministry of Public Health (MINSAP), and reports from international organizations (WHO, PAHO, IAHPC, ALCP). Two authors independently screened identified records; disagreements were resolved by discussion with a third author. The WHO analytical framework for PPC integration was applied to evaluate six key dimensions: policy, education, medicines, services, research, and financing.

**Results:**

Cuba has a solid normative basis for PPC, including Ministerial Resolution No. 261/2020 and the National Cancer Control Program. Primary care coverage is universal, and the family doctor system facilitates care continuity, though the current level of formal PC training among family physicians specifically has not been systematically documented. Gaps were identified in: specialized staff training, equitable access to opioids at home, formal constitution of PPC teams in all eight centers, and published clinical research on outcomes in this population.

**Conclusions:**

Cuba has favorable structural conditions for implementing an integrated, high-quality PPC model for children with cancer. The implementation of the National Pediatric Palliative Care Project 2026–2028 represents an important opportunity. Explicit alignment with the WHO model, strengthening specialized training, and generation of national scientific evidence are recommended.

## Background

Cancer in the pediatric age group (0–18 years) affects more than 400,000 children and adolescents annually worldwide, of whom approximately 90% live in low- and middle-income countries where access to timely diagnosis, oncological treatment, and palliative care (PC) is limited (1). In Cuba, the estimated incidence ranges between 350 and 430 new cases per year, with a rate of approximately 10–14 per 100,000 children under 15 years of age, and cancer represents the second leading cause of death in that age group, after accidents (2).

Pediatric palliative care (PPC) is recognized by the WHO as an essential component of oncological care that should be integrated from the moment of diagnosis, in parallel with curative-intent treatment (3). Evidence accumulated over the past two decades, largely from adult oncology populations, suggests that early integration improves symptom control and quality of life for both patient and family, and may reduce disproportionate end-of-life interventions (4, 5). Comparable pediatric-specific trial evidence remains limited.

Cuba has unique characteristics that distinguish it from other countries in the Americas regarding PPC provision: a universal and free health system with 100% population coverage, a national network of family physicians present in every community, eight pediatric oncology reference centers distributed across the main provinces, and a regulatory framework that recognizes PC as a citizen's right. However, the systematic and equitable implementation of specialized PPC faces structural, educational, and cultural challenges that this review aims to analyze.

This review focuses specifically on pediatric oncology, given that Cuba's PPC-relevant infrastructure, regulatory framework, reference-center network, and national palliative care project are all structured around this population; the PPC needs of children with non-oncological life-threatening or life-limiting conditions (e.g., neurological, metabolic, cardiac, or other complex chronic disease) fall outside the scope of this review and warrant separate, dedicated investigation.

The objectives of this article are to describe the state of PPC development for children with oncological disease in Cuba according to the WHO simultaneous integration model, identify existing gaps, and propose a set of evidence-based recommendations for its strengthening within the context of the Cuban health system.

## Methods

A narrative review of the scientific literature indexed in PubMed/MEDLINE, LILACS, and SciELO, published between January 2000 and December 2024, was conducted using the following search terms: “pediatric palliative care,” “childhood cancer palliative care,” “Cuba palliative care,” “Latin America pediatric palliative care,” “WHO palliative care model,” and their Spanish equivalents. Original articles, systematic reviews, narrative reviews, position documents, and clinical guidelines from relevant international organizations were included.

Titles and abstracts of records identified through the search strategy were screened independently by two authors (MFS and DCQV) for relevance; full texts of potentially eligible records were then assessed independently by the same two reviewers. Disagreements regarding inclusion were resolved through discussion, with a third author (MCLA) consulted where consensus could not be reached. As this is a narrative rather than a systematic review, formal risk-of-bias assessment and a PRISMA-type flow diagram were not applied; the search strategy and inclusion approach are reported here for transparency rather than as a claim of systematic-review methodology.

Additionally, normative documents of the Cuban Ministry of Public Health (MINSAP) were reviewed, including Ministerial Resolution No. 261/2020 on palliative care, the current National Cancer Control Program, the guidelines of the National Pediatric Oncology Working Group, and applicable health legislation. Situation reports from the Pan American Health Organization (PAHO), the Latin American Association for Palliative Care (ALCP), and the International Association for Hospice and Palliative Care (IAHPC) were also consulted.

This review was limited to pediatric oncology because Cuba's existing regulatory framework, reference center network, and national palliative care project (MINSAP-PRCP-OPD-2025-001) are structured specifically around this population.

The WHO analytical framework for PPC integration in pediatrics (WHO, 2018) (3) was applied to evaluate six key dimensions: (1) policy and regulation, (2) education and training, (3) access to essential medicines, (4) services and provision models, (5) research and evidence generation, and (6) financing and sustainability.

## The WHO model of pediatric palliative care integration

### Foundations of the model

The WHO published its first definition of PPC in 1998, updating the concept in 2018 in its document “Integrating palliative care and symptom relief into pediatrics” (3). This document represents the international reference standard for the design and evaluation of PPC programs at the national and subnational levels.

The WHO model rejects the traditional conception that placed PC exclusively in the terminal stage of illness, defining it as “the active and total care of the child’s body, mind and spirit, and involves giving support to the family,” with initiation from the diagnosis of a life-threatening or life-limiting condition, in parallel with active treatment and continuing through the bereavement period (3).

The central concept of the model is simultaneous integration: PPC does not replace active oncological treatment but complements it from day one, with the objective of maximizing the quality of life for patient and family regardless of prognosis and therapeutic goal (5). Randomized clinical trials in adult oncology populations have shown favorable outcomes from this integration, including improved symptom control and quality of life, reduced use of emergency and hospital services, and greater patient and family satisfaction with care (4, 6); pediatric-specific randomized evidence for these outcomes remains scarce.

### The four categories of children with PPC needs (ACT/RCPCH classification)

The WHO model adopts the classification of the Association for Children's Palliative Care and the Royal College of Pediatrics and Child Health, which groups children with PPC needs into four categories according to the type of condition and prognosis (7) (Table 1).

**Table 1:** ACT/RCPCH categories of children with PPC needs adopted by the WHO model.

Category	Description	Oncological examples	Implication for PPC
I	Condition with treatment that may fail; possibility of cure	High-risk leukemia; Wilms tumor stage IV	Preventive and supportive PPC during active treatment
II	Disease in which premature death is inevitable, although survival may be prolonged	Unresectable low-grade brain tumor	Continuous PPC integrated with oncological treatment
III	Progressive condition without curative treatment; PC as the only active treatment	Pediatric glioblastoma; DIPG	Predominantly specialized PPC; home care
IV	Irreversible non-progressive condition with risk of premature death from complications	Severe oncological sequelae; posttransplant multiorgan failure	Maintenance PPC and long-term follow-up

## Results

The following section presents the analysis of each dimension of the WHO model in the context of the Cuban health system, identifying advances achieved, existing gaps, and opportunities for improvement, within the pediatric oncology population.

### Dimension 1: policy and regulation

Cuba has a legal and regulatory framework that explicitly recognizes palliative care as a citizen's right. The Constitution of the Republic of Cuba (2019) establishes in Articles 90 and 92 the right to health and special protection of childhood. The Family Code (2022) recognizes children's rights to adequate and comprehensive medical care. Ministerial Resolution MINSAP No. 261/2020 on palliative care in the National Health System represents the most relevant regulatory milestone: it recognizes PC as an essential component of health care and mandates its incorporation in health services.

The National Cancer Control Program includes specific guidelines for pediatric oncological care, and the Special Pediatric Oncology Working Group acts as the governing body for sector policy. Cuba ratified the UN Convention on the Rights of the Child (Article 24: right to the highest attainable standard of health), reinforcing the international regulatory framework.

However, the analysis identified relevant regulatory gaps: (a) Resolution No. 261/2020 does not specify minimum structural or process standards for specialized PPC; (b) there is no specific regulation on home prescription of opioids facilitating access for pediatric oncology patients at home; (c) advance care planning (ACP) and the concept of advance directives lack regulatory development in Cuban legislation; and (d) the concept and implementation of progressive autonomy is still under development.

- Favorable constitutional and legal framework (Constitution 2019, Family Code 2022)
- Ministerial Resolution No. 261/2020: explicit recognition of PC
- National Cancer Control Program with pediatric oncology component
- Gap: no specific minimum standards for specialized PPC
- Gap: no specific regulation for pediatric home opioid access
- Gap: no regulatory framework for advanced directives and progressive autonomy in pediatrics

### Dimension 2: education and training

PC training for Cuban health personnel caring for children with cancer is one of the areas with the greatest gap relative to the WHO model. Although the National School of Public Health (ENSAP) incorporates PC content in some postgraduate programs, there is currently no medical residency or formal subspecialty in PPC in Cuba. Most pediatric oncologists and nurses caring for pediatric oncological patients lack structured training in basic PC competencies. Only one general PC diploma exists, without specific pediatric training. The training plans for the specialties of Medical Oncology, Surgical Oncology, and Radiotherapy include a PC module that addresses PPC (8).

Family physicians occupy a central role in the care-continuity model discussed later in this review (Dimension 4), yet the current level of formal palliative or pediatric palliative care training specifically among family physicians has not been systematically documented in Cuba. Their curricula include general components of chronic disease management and home care, but structured PC-specific competency training comparable to that offered in hospital-based oncology specialties does not currently exist at the primary care level. Closing this documentation and training gap is identified as a priority for the National PPC Project.

The 2025 global mapping of palliative care development, conducted under the updated WHO framework across 201 countries and territories, situates Cuba within an intermediate development band for palliative care provision, superseding the earlier 2020 regional atlas classification and highlighting insufficient specialized professional training as a main obstacle (9). Regional studies conducted across Latin America suggest that a substantial proportion — in the range of 60–75% in some national samples — of medical and nursing staff in pediatric oncology services report not having received formal PC training during undergraduate education or specialization (10); this figure derives from regional, not Cuba-specific, data and should not be interpreted as a national estimate for Cuba.

Nevertheless, the Cuban health system presents notable educational strengths: high physician density per capita (more than 8 physicians per 1,000 inhabitants), a culture of continuing medical education through teaching polyclinics, and the existence of distance education platforms such as Infomed that could be used for mass training in basic PC competencies.

The National PPC Project 2026–2028 plans training of 240 professionals in basic competencies (Level 1), 80 in advanced competencies (Level 2), and 16 team coordinators in a PPC diploma (Level 3), following the WHO-recommended cascade training model (11).

- High physician density and culture of continuing education
- Educational infrastructure: ENSAP, Infomed, teaching polyclinics
- Cascade training plan included in National Project 2026–2028
- Gap: no medical residency or formal subspecialty in PPC
- Gap: insufficient and non-standardized undergraduate and postgraduate PC training
- Gap: training status of family physicians in PC/PPC not systematically documented
- Regional estimate (not Cuba-specific): 60–75% of pediatric oncology staff without formal PC training

### Dimension 3: access to essential medicines

Access to essential medicines for pain relief and other symptoms is a key indicator of PC development. The WHO considers oral and parenteral morphine to be the reference opioid in PPC, and its availability is an indispensable condition for ensuring care quality (3). Cuba includes morphine in its National Essential Medicines List, representing an important regulatory advance compared to other countries in the region. Recent international literature has highlighted persistent, widespread inequities in children's access to controlled medicines for pain relief globally, underscoring that this is not a challenge unique to Cuba but a broadly documented gap in pediatric palliative care worldwide (12).

However, analysis of the literature and available data identifies relevant gaps in effective access to opioids in the Cuban pediatric oncology context: (a) morphine prescription requires a special control prescription with a bureaucratic circuit that may delay access in emergency situations; (b) home access to opioids for pediatric patients in advanced PC depends on coordination between the hospital team, family physician, and local pharmacy, without a standardized national protocol; and (c) certain pediatric formulations (e.g., low-dose fentanyl patches) are not available.

In the Latin American context, per capita morphine consumption is an indicator of PC access: Cuba shows morphine consumption of 0.2–0.5 mg per capita/year, significantly lower than countries with welldeveloped PC (Germany: 85 mg; USA: 695 mg; Uruguay: 12 mg), although comparable to other countries in the region with similar development levels (13). Table 2 summarizes the availability and identified gaps for the main essential medicines used in PPC in Cuba.

- Morphine included in the National Essential Medicines List
- Essential PC medicines are mostly available in hospitals
- Free health system: no economic barrier to access
- Gap: low per capita morphine consumption relative to international standards
- Gap: bureaucratic opioid prescription circuit may delay access
- Gap: home access to pediatric opioids without standardized national protocol
- Gap: specific pediatric formulations with irregular availability

**Table 2:** Availability and access gaps for main paediatric palliative care medicines in Cuba.

Essential PC medicine	Availability in Cuba	Identified gap
Oral morphine (solution)	Essential list, not consistently available	Bureaucratic circuit; no standardized pediatric formulation
Injectable morphine SC/IV	Available in hospitals	Home access dependent on local coordination
Injectable midazolam	Hospitals; limited in primary care	No anticipatory prescription for home use in PPC
Transdermal fentanyl	Not available	Not available
Oral methadone	Specialized hospital use	No protocol for home PPC uses
Gabapentin	Essential list	Variable availability in some provinces
IV dexamethasone	Widely available	No significan gap identified
IV/oral ondansetron	Available in hospitals	Variable outpatient access
SC haloperidol	Essential list	No significant gap in hospitals

### Dimension 4: services and provision models

Cuba has eight pediatric oncology reference centers that concentrate the diagnosis and treatment of children with cancer nationally. These centers, located in major cities (three in Havana, one each in Pinar del Río, Villa Clara, Camagüey, Holguín, and Santiago de Cuba), have different levels of PPC development.

Analysis of the current situation reveals that, unlike health systems with well-developed PPC where interdisciplinary PC teams work in parallel with the oncology team from diagnosis, in Cuba PC care in pediatric oncology is predominantly reactive, mono-sectoral (centered on the oncologist), and primarily oriented toward the end-of-life phase. Of the eight oncology reference centers, only the Institute of Oncology and Radiobiology (INOR) has a formally constituted PPC team with the minimum components recommended by the WHO (physician, nurse, psychologist, social worker).

Cuba possesses two structural strengths that may support development of an integrated PPC model with care continuity: (a) family physicians and nurses present in every community, who know the patient and family and can support continuity of home care; and (b) a tradition of teamwork in polyclinics, with basic work groups including physician, nurse, psychologist, and social worker — a structure that aligns closely with the WHO-recommended interdisciplinary PPC model.

- Network of 8 pediatric oncology reference centers with national coverage
- Family physician system: potentially valuable structure for care continuity in PPC
- Primary care basic work groups aligned with the WHO interdisciplinary model
- Gap: no formal PPC teams in 7 of the 8 oncology centers to date
- Gap: reactive care centered on the terminal phase, not integrated from diagnosis
- Gap: no standardized hospital-home continuity model for PPC

### Dimension 5: research and evidence generation

Cuban scientific production on pediatric oncology PPC is scarce. A search in PubMed and LILACS using the terms “palliative care” AND “Cuba” AND (“pediatric” OR “children”) yielded fewer than 15 original articles published between 2000 and 2024, none corresponding to a clinical trial or cohort study reporting quality of life outcomes in pediatric oncological patients receiving PC. Most publications found are narrative reviews, case reports, or position articles.

This scarcity of national scientific evidence has relevant practical consequences: it hinders adaptation of international guidelines to the Cuban reality, limits rigorous evaluation of the impact of PPC interventions in the country's pediatric oncology population, and reduces the international visibility of Cuba's experience in this area. It also constrains Cuba's capacity to contribute to the Latin American body of evidence on PPC, which also shows deficiencies compared to evidence from Europe and North America (12).

- Network of centers with potential for multicenter research
- National Cancer Registry as a source of population data
- Publication plan included in National Project 2026–2028
- Gap: very scarce national scientific production on PPC (< 15 articles 2000–2024)
- Gap: no clinical trials or cohort studies on pediatric PC outcomes in Cuba
- Gap: no specific funding for PPC pediatric oncology research

### Dimension 6: financing and sustainability

The Cuban health system is public and free, financed by the State through the national budget. This characteristic eliminates one of the main barriers to PPC access in low- and middle-income countries: cost to patient and family. However, scarcity of material resources and the budget planning system represent challenges for specific resource allocation for PPC.

There is currently no specific budget line for PPC within the MINSAP or hospital budgets. Resources allocated to this activity come from the general Pediatric Oncology budget, generating competition with other service priorities. International cooperation, particularly from PAHO, IAHPC, and ALCP, has provided technical and training support, but there is no formal and sustainable external financing mechanism for PPC in Cuba.

- Free health system: no economic barrier for patients and families
- Technical cooperation from PAHO, IAHPC, and ALCP available
- Gap: no specific budget line for PPC
- Gap: resources compete with other oncology budget priorities
- Gap: post-project sustainability not guaranteed without regulatory budget change

### Synthesis of PPC development in Cuba according to the WHO model

Table 3 presents a cross-dimensional synthesis of the main advances, gaps, and development level identified for each of the six WHO dimensions in the Cuban pediatric oncology context.

**Table 3:** Synthesis: six WHO dimensions applied to Cuba’s pediatric oncology PPC reality.

WHO dimension	Main advances	Main gaps	Development level
1. Policy & Regulation	Constitution 2019; Res. 261/2020; National Cancer Program; UN Convention ratification	No specific PPC standards; no pediatric home opioid regulation; no advance directives framework	Moderate — solid legal basis, partial implementation
2. Education & Training	High physician density; ENSAP; Infomed; 2026–2028 training plan	No PPC residency/subspecialty; insufficient training; family physician PPC training undocumented	Initial — improvement plan underway
3. Medicines Access	Morphine on essential list; free system; medicines available in hospitals	Low per capita consumption; home access not protocolized; irregular pediatric formulations	Moderate — regulatory availability, limited effective access
4. Services & Models	8 oncology reference centers; family physician network; primary care basic work groups	No formal PPC teams in 7 of the 8 oncology centers; reactive care; no standardized hospital-home model	Initial — favorable structure without formal implementation
5. Research	Cancer registry; multicenter potential; publication plan 2026– 2028	< 15 original articles 2000–2024; no clinical trials; no research funding	Initial — potential capacity without systematic production
6. Financing	Free public system; international technical cooperation	No specific budget line; post-project sustainability not guaranteed	Incipient — undifferentiated financing for PPC

## Discussion

### Cuba in the Latin American context

PPC development for children with cancer in Cuba is comparable to Latin American countries with universal health coverage systems (Costa Rica, Uruguay, Ecuador), but lags countries with more consolidated PPC programs such as Argentina, Brazil, and Chile. The latter have formally constituted PPC teams in their main pediatric oncology centers, recognized academic training programs, and more systematic scientific production (12). However, Cuba compares favorably with most countries in the region in terms of primary care coverage, equity of access, and the existence of a health system that eliminates the economic barrier.

The experience of Uruguay, a country with higher per capita GDP but with similarities in health system size and medical culture, is a relevant reference point. Uruguay implemented a national PC program including PPC in 2013, with specific budget allocation and mandatory PC training for family physicians and pediatricians. Ten-year results reported a reduction in time from diagnosis to first PC assessment and an increase in the percentage of deaths occurring in the family's preferred location (14).

### The family physician model as a potential structural advantage

A distinctive characteristic of the Cuban system relevant to PPC development is the national network of family physicians and nurses. With more than 30,000 family physicians distributed in community clinics throughout the country, Cuba has a human resource base that few other Latin American health systems possess at the same scale: a health professional who typically knows the child and family from before the cancer diagnosis, has home access, and can support continuity of care in the community. As noted in Dimension 2, however, the extent to which this workforce currently holds formal PC/PPC competencies has not been systematically documented, and this should be treated as an open question rather than an assumed strength.

This resource may be particularly relevant for supporting home-based PPC, which international evidence indicates as a preferred setting for many children in advanced stages, and a preferred place of death for most families in some studies (15, 16). Implementation of a structured hospital-home continuity model capitalizing on the family physician figure could represent a potentially innovative and exportable aspect of a future Cuban PPC model, contingent on closing the training and protocol gaps identified above.

### Cultural and attitudinal barriers

Beyond the structural barriers analyzed, the literature identifies cultural and attitudinal barriers affecting PPC development in Cuba, like those described in other countries in the region. These include unfounded fear of opioids (opiophobia) among prescribers and families; the perception that initiating PC equates to “giving up” or “abandoning the patient”; difficulty communicating adverse prognostic information in the Cuban cultural context; and resistance to early PC integration in services oriented toward active treatment (10, 17).

These barriers can potentially be modified through training, awareness, and institutional leadership. International experience suggests that cultural change in oncological services toward PC integration is achievable over a period of several years when there is political will, a structured training plan, and committed clinical leaders (5).

### Research as a lever for change

The scarcity of national scientific evidence on pediatric oncology PPC in Cuba is not merely an academic problem: it constitutes a barrier to data-driven decision making, adaptation of international guidelines to local reality, and evaluation of intervention impact. Evidence generation may be one of the most useful instruments for advancing PPC development: quality-of-life data, pain control outcomes, and family satisfaction data obtained in the Cuban context could serve as effective arguments for justifying resource investment and regulatory reform.

The National PPC Project 2026–2028 should incorporate a prospective evaluative research component to document program impact from its outset. Creation of a national pediatric oncology PPC database, linked to the National Cancer Registry, would represent a scientific and policy asset of value for the Cuban health system.

### Scope and limitations

This review has several limitations. First, as a narrative rather than systematic review, it does not include a formal risk-of-bias assessment or exhaustive database coverage. Second, several quantitative estimates cited (e.g., regarding staff training gaps) derive from regional Latin American literature rather than Cubaspecific studies and should be interpreted accordingly. Third, and most importantly, this review is limited to children with oncological conditions and does not address PPC needs of children with other lifethreatening or life-limiting illnesses (e.g., severe neurological impairment, congenital or metabolic disease, complex chronic conditions), who represent a substantial share of the pediatric population requiring palliative care globally. Future work should evaluate the applicability of the WHO integration framework beyond oncology in the Cuban context.

## Conclusions

Cuba has structural, regulatory, and cultural conditions that place it in a favorable position for developing an integrated, equitable, and high-quality PPC model for children with cancer, aligned with WHO recommendations. The universal and free health system, the family physician network, the existing regulatory framework, and the National PPC Project 2026–2028 represent a foundation on which to build.

The gap between the regulatory framework and actual implementation remains significant. The constitution of formal PPC teams in the eight oncology reference centers, systematic health personnel training (including documentation and strengthening of family physician competencies), protocolization of home opioid access, and generation of national scientific evidence are among the most pressing priorities to advance from an “initial” to a more integrated development level according to the WHO classification.

The family physician model may represent one of Cuba's more distinctive structural advantages for PPC development in oncology; its strategic capitalization in a hospital-home care continuity model could potentially contribute an original model of good practice for PPC in resource-limited settings, pending the training and evidence-generation work identified in this review.

Rigorous implementation of the National Project 2026–2028, with systematic indicator monitoring and publishable evidence generation, may not only improve care for Cuban children with cancer and their families, but could also help position Cuba as a relevant Latin American reference in pediatric oncology palliative care.

### Recommendations for strengthening PPC in Cuba

Based on the gap analysis presented above, Table 4 summarizes the evidence-based strategic recommendations for PPC strengthening in Cuban pediatric oncology, organized by WHO dimension, proposed timeline, and lead responsible entity.

**Table 4:** Strategic recommendations for PPC strengthening in Cuban pediatric oncology, aligned with the WHO model.

Dimension	Recommendation	Timeline	Lead responsible
Policy	Develop specific PPC regulations establishing minimum standards for the 8 oncology centers	Short (2026–2027)	MINSAP — Pediatric Oncology Working Group
Policy	Establish a national protocol for pediatric home opioid prescription and access	Short (2026)	MINSAP — Pharmacy and Optics Cuba
Policy	Develop a Cuban regulatory framework for ACP in pediatrics	Medium (2027– 2028)	MINSAP — National Bioethics Commission
Education	Document current PC/PPC training levels among family physicians and incorporate mandatory PPC modules across pediatric oncology, pediatrics, and general medicine specialties	Medium (2027– 2028)	Ministry of Higher Education — ENSAP
Education	Design and implement a recognized postgraduate PPC residency or master’s degree	Long (2029–2031)	MINSAP — Medical Sciences Universities
Medicines	Develop standardized pediatric morphine oral formulations and ensure national availability	Short (2026–2027)	MINSAP — LABIOFAM —Pharmacy and Optics Cuba
Services	Formally constitute PPC teams in all 8 oncology reference centers	Immediate (2026)	Hospital directors — National Working Group
Services	Implement a standardized hospital-home continuity model integrating the family physician as a key actor	Short (2026–2027)	National Working Group — Provincial Primary Care
Research	Create a prospective national pediatric oncology PPC database linked to the National Cancer Registry	Short (2026–2027)	National Working Group — MINSAP — INOR
Financing	Include a specific PPC budget line in the health budget of the 8 provinces with reference centers	Medium (2027– 2028)	MINSAP — Ministry of Finance
